# Cyclodextrin-Modified Capillary Zone Electrophoresis for the Chiral Analysis of Proline and Hydroxyproline Stereoisomers in Chicken Collagen Hydrolysates

**DOI:** 10.3390/ijms26125832

**Published:** 2025-06-18

**Authors:** Milada Vodova, Elena Babini, Francesca Soglia, Martina Bordini, Martina Lioi, Sara Tengattini, Caterina Temporini, Roberto Gotti

**Affiliations:** 1 Department of Chemistry and Biochemistry, Mendel University in Brno, Zemedelska 1, 61300 Brno, Czech Republic; vodova.milada@seznam.cz; 2 Department of Agricultural and Food Sciences (DISTAL), University of Bologna, 47521 Cesena, Italy; elena.babini2@unibo.it (E.B.); francesca.soglia2@unibo.it (F.S.); martina.bordini5@unibo.it (M.B.); 3 Department of Drug Sciences, University of Pavia, Viale Taramelli 12, 27100 Pavia, Italy; martina.lioi01@universitadipavia.it (M.L.); sara.tengattini@unipv.it (S.T.); caterina.temporini@unipv.it (C.T.); 4 Department of Pharmacy and Biotechnology, University of Bologna, Via Belmeloro 6, 40126 Bologna, Italy

**Keywords:** capillary electrophoresis, hydroxyprolines, chiral separations, collagen, cyclodextrins, derivatization, fluorescent detection, fast-growing chickens, wooden breast, spaghetti meat

## Abstract

The stability of collagen, the most abundant protein in humans and many animals, is related to the hydroxylation of L-proline, a post-translational modification occurring at carbon 3 and 4 on its pyrrolidine ring. Collagens of different origins have shown different proline hydroxylation levels, making hydroxyprolines useful biomarkers in structure characterizations. The presence of two chiral carbon atoms, 3-hydroxyproline and 4-hydroxyproline, results in eight stereoisomers (four pairs of enantiomers) whose quantitation in collagen hydrolysates requires enantioselective analytical methods. Capillary electrophoresis was applied for the separation and quantitation of the eight stereoisomers of 3- and 4-hydroxyproline and D,L-proline in collagen hydrolysates. The developed method is based on the derivatization with the chiral reagent (R)-(-)-4-(3-Isothiocyanatopyrrolidin-yl)-7-nitro-2,1,3-benzoxadiazole, enabling the use of a light-emitting diode-induced fluorescence detector for high sensitivity. The separation of the considered compounds was accomplished in less than 10 min, using a 500 mM acetate buffer pH 3.5 supplemented with 5 mM of heptakis(2,6-di-O-methyl)-β-cyclodextrin as the chiral selector. The method was fully validated and showed the adequate sensitivity for the application to samples of collagen hydrolysates. The analysis of samples extracted from chicken *Pectoralis major* muscles affected by growth-related myopathies showed different stereoisomer patterns compared to those from the unaffected control samples.

## 1. Introduction

**Figure 1:**
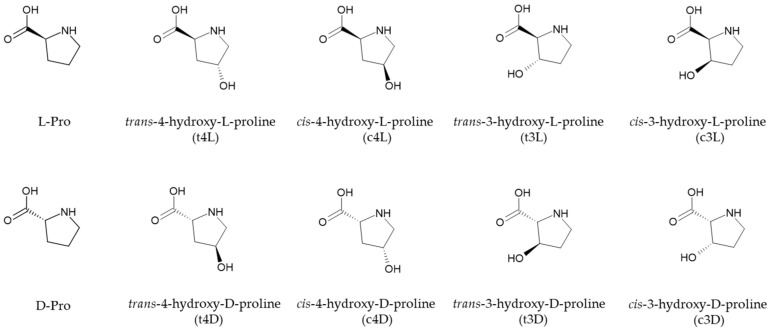
The structure of proline and hydroxyproline stereoisomers and the related abbreviations used in this paper.

Collagen is a fundamental constituent of connective tissues and the most abundant protein in humans and many animals. Structurally, collagen consists of three polypeptide chains, and the α-chains are each constituted of a repeating triplet of amino acids (i.e., glycine, proline, and hydroxyproline); this repeating pattern is essential for stabilizing the structure of the triple helix [1]. Prolyl hydroxylases enzymes are responsible for the post-translational modification at the carbons in position 3 and 4 on the pyrrolidine ring of L-proline, producing *trans*-3-hydroxy-L-proline and *trans*-4-hydroxy-L-proline, respectively, occurring in the collagen of many animal species with a ratio of about 1:100 [2]. The proper hydroxylation of L-proline residues is critical for the homeostasis of tissues, as this modification imparts both stability and structural integrity to collagen [3,4]. In human fibrillar collagen the proline hydroxylation ranges within 42–54%, whereas the levels can be significantly lower in recombinant collagen [5]. In connective tissue diseases, prolyl hydroxylation aberrations alter the hydroxylation levels, changing the quality of the collagen molecules [6]. Thus, assessing the L-proline hydroxylation level by quantifying the hydroxyproline stereoisomers in collagen and its variants is essential. Both 4-hydroxyproline and 3-hydroxyproline possess two chiral carbon atoms, resulting in a total of eight stereoisomers, i.e., four enantiomer pairs. The restricted rotation of the carbon skeleton of the pyrrolidine ring bearing the hydroxy and carboxylic functions results in *cis*/*trans* stereoisomers (Figure 1).

Unlike *trans*-4-hydroxy-L-proline and *trans*-3-hydroxy-L-proline, the other hydroxyproline isomers are not represented in animal tissues under physiological conditions. The acid hydrolysis, carried out to release the amino acids for the compositional analysis of collagen samples, has been shown to cause chemical isomerization, yielding small amounts of *cis*-4-hydroxy-D-proline and *cis*-3-hydroxy-D-proline [7]. Interestingly, certain animal tissues contain proline epimerases capable of converting the hydroxyproline isomers commonly found in collagen into their stereoisomers [2]. Therefore, chiral methods for the analysis of the complete series of hydroxyproline isomers would be highly valuable in studies concerning their biological and pathophysiological significance. A pioneering study on the separation of hydroxyproline stereoisomers was published by Langrock et al. based on the chiral derivatization of the target analytes with the reagent Nα-(2,4-dinitro-5-fluorophenyl)-L-valinamide. The proposed method enabled the HPLC separation of six stereoisomers (among them, two enantiomer pairs) and their quantitation in commercially available collagen samples after gas-phase acid hydrolysis [8]. We recently reported a study utilizing the same chiral reagent, and by means of a reversed-phase HPLC stationary phase, the complete separation of all eight stereoisomers (the four enantiomer pairs) was achieved using isocratic elution and an MS-compatible mobile phase. The method was applied to collagen hydrolysates from different species and to recombinant human type II collagen, showing significant differences in the content of hydroxyproline stereoisomers, which were found in agreement with the consensus sequences in the primary chain [9]. Gas chromatography (GC) has been successfully applied to the separation of some of the hydroxyproline stereoisomers. In an early study by Lüpke and Brückner, *cis*-4-hydroxy-D-proline and *trans*-4-hydroxy-L-proline were separated in a single run, simultaneously with the chiral resolution of D,L-Proline [10]. More recently Opekar et al. proposed a GC-MS method that allowed the chiral separation of D,L-Proline and the 3- and 4-hydroxyproline stereoisomers, with the exception of *trans*-4-hydroxy-L-proline and *cis*-4-hydroxy-D-proline. The method was applied to different samples, including collagen hydrolysates [11]. Capillary electrophoresis (CE) is a technique of choice in chiral analysis due to its ability in achieving a chiral resolution by combining a high efficiency and wide opportunity for the enantioselectivity tuning, by directly supplementing the electrophoretic buffer (background electrolyte, BGE) with the chiral selectors [12]. Having the chiral selector as a component of the BGE allows transient diastereomers to be formed by the interaction with the analytes during their electrophoretic run. Cyclodextrins (CDs) are among the most used chiral selectors for their ability to establish intermolecular noncovalent interactions, often based on the inclusion complexation of the analytes into the CD cavity [13,14]. Many applications of CDs as chiral selectors in CE focus on the pharmaceutical field for drug chiral purity control [15]. CDs can be employed either as the sole chiral selector (alone or in dual CDs mixtures) or to enhance the enantioresolution in conjunction with the chiral derivatization of the analytes, as well as in the presence of other chiral selectors dissolved into the BGE or immobilized onto the inner silica wall [15,16]. The enantioseparation of hydroxyproline stereoisomers by CE has been scarcely explored and limited to some of the compounds shown in Figure 1. Phenylisothiocyanate derivatives of 4-hydroxyproline (*cis* and *trans*) and 3-hydroxyproline have been separated by micellar electrokinetic chromatography (MEKC) using a coated capillary; the method, not addressing the enantiomer separation, was applied to collagen hydrolysates [17]. More recently, the chiral separation of the two enantiomer pairs of 4-hydroxyproline has been achieved by capillary zone electrophoresis (CZE) after the derivatization of the analytes with 9-fluorenylmethylchloroformate (FMOC-Cl), using methyl-γ-cyclodextrin as the chiral selector. The application was focused on the determination of the free 4-hydroxyproline stereoisomers in cosmetic samples [18].

In the present study, the separation of the complete series of 3- and 4-hydroxyproline stereoisomers, including D,L-proline (Figure 1), was achieved by means of a cyclodextrin-modified CZE (CD-CZE) method. Compared to our previous HPLC-based separation [9], the CD-CZE approach offered a significantly faster analysis and represents a valuable orthogonal method for comparative and routine applications. To achieve the desired selectivity and sensitivity, the analytes were derivatized with the chiral reagent (R)-(-)-4-(3-Isothiocyanatopyrrolidin-yl)-7-nitro-2,1,3-benzoxadiazole ((R)-NCS), enabling the use of a light-emitting diode-induced fluorescence (LEDIF) detector. The resolution of the compounds was obtained by the addition of either heptakis(2,6-di-O-methyl)-β-cyclodextrin (DM-βCD) or β-cyclodextrin (βCD) to the BGE, showing the usefulness of the CDs as additives and constituents of the buffer for tuning the selectivity of complex enantiomer mixtures. The method was validated and applied to the determination of hydroxyproline and proline stereoisomers in collagen type III extracted from chicken *Pectoralis major* muscles affected by growth-related abnormalities.

## 2. Results and Discussion

The separation and quantitation at the analytical scale of proline and hydroxyproline stereoisomers, recognized as highly specific and valuable markers in the characterization of collagen hydrolysates, was addressed by CE due to its high resolution, minimal sample and reagent consumption, and for the established utility in chiral analytical separations.

### 2.1. The Optimization of the Separation Conditions

**Figure 2:**
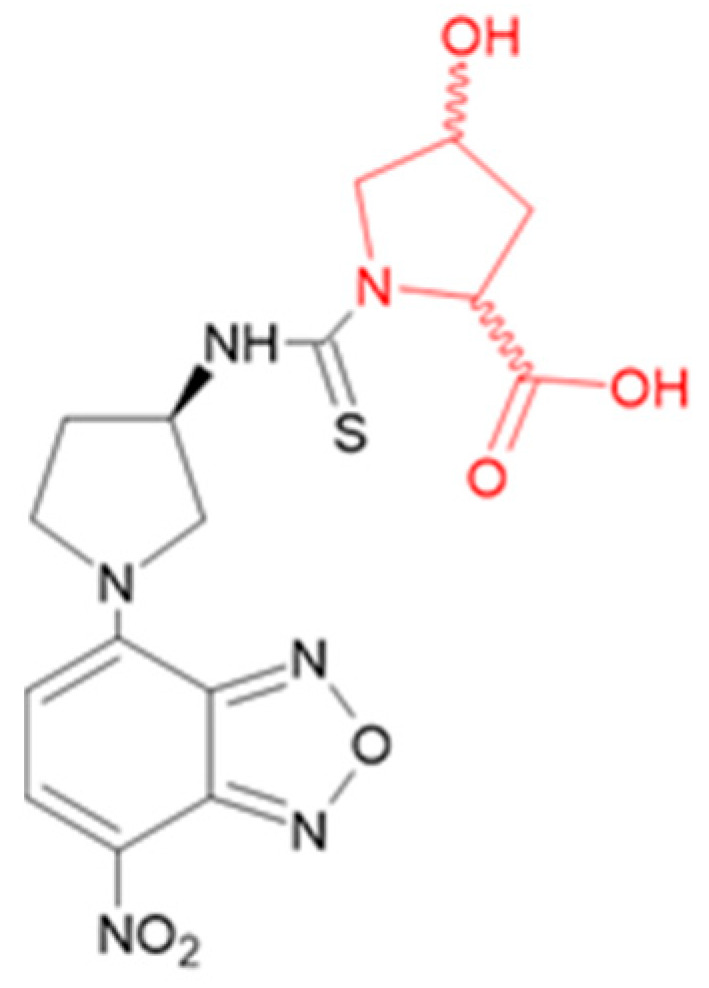
The proposed structure of the thiourea derivative [19] obtained by the reaction of the derivatization reagent (R)-NCS (black moiety) with a generic 4-hydroxyproline (red moiety).

The separation of proline and hydroxyproline stereoisomers for determinations in collagen hydrolysates is challenging as it requires both chemo- and stereoselectivity to differentiate the closely related target analytes within a complex matrix containing all the other amino acids released from the protein. Furthermore, except for L-Pro and t4L, the target solutes are expected to be at low concentrations, requiring a high detection sensitivity. LEDIF-oriented derivatization using the fluorescent Edman-type reagent (R)-NCS was selected to improve both the selectivity and detection sensitivity. This reagent quantitatively reacts with amino functions, forming stable thiourea derivatives with a fluorescence excitation wavelength at 480 nm, which is compatible with common LEDIF detectors [19,20]. According to the proposed structure (Figure 2), the produced derivatives keep the carboxylic function of the original amino acids as the sole ionizable center. The pKa values of the carboxy group of the proline and hydroxyprolines are reported to be in the range of 1.6–2.0, thus BGEs with a pH below four were considered, as under this condition the studied compounds undergo partial ionization and dissociate to different extents, allowing for the maximized selectivity in CZE. On the other hand, at pH values below two, a too weak electroosmotic flow (EOF) resulted in the low apparent mobility of the derivatives with long migration times.

**Figure 3:**
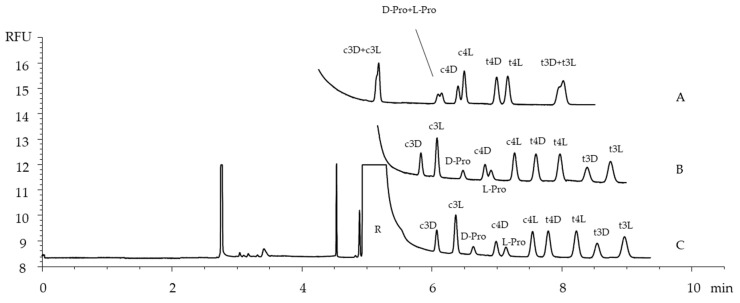
Electropherograms of the separation of D,L-Pro and hydroxyproline amino acids derivatized with (R)-NCS using a BGE composed of 500 mM acetate buffer with a pH of 3.5 (**A**), supplemented with DM-βCD at 2.5 mM (**B**) and 5 mM (**C**). Other conditions: a fused-silica capillary with a 55.5 cm total length (34 cm, length to the detector); 50 μm i.d.; voltage 30 kV; temperature 25 °C; hydrodynamic injection at 50 mbar × 10 s; and LEDIF detection (480 nm excitation wavelength). Symbols: Reagent excess (R); the peaks are the thiourea derivatives of the compounds whose abbreviations are given in Figure 1. In the electropherograms (**A**,**B**) the peak of the reagent excess (R) has been erased.

The acetate buffer is suitable for the preparation of BGEs in the selected pH range; a good compromise between the analysis time and resolution was obtained at a pH of 3.5. Despite the diastereomeric nature of the derivatives, their complete separation in the achiral environment was not achieved (Figure 3A). Native CDs, such as β-cyclodextrin (βCD) and γ-cyclodextrin (γCD), as well as derivatized neutral CDs, i.e., (2-hydroxypropyl)-β-cyclodextrin (HP-βCD), heptakis(2,6-di-O-methyl)-β-cyclodextrin (DM-βCD), and heptakis(2,3,6-tri-O-methyl)-β-cyclodextrin (TM-βCD), were used as additives to the BGE to improve the enantioresolution.

**Figure 4:**
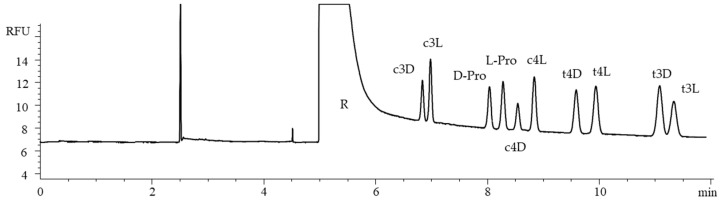
The electropherogram of the separation of D,L-Pro and hydroxyproline amino acids derivatized with (R)-NCS using a BGE composed of a 500 mM acetate buffer with a pH of 3.5 supplemented with βCD at a 5 mM concentration. Other conditions and symbols as in Figure 3.

βCD and DM-βCD when individually supplemented to the BGE exhibited the highest chemo- and stereoselectivity which could be optimized by properly adjusting their concentration as the buffer components. The effect of the DM-βCD level on the separation of the considered compounds is shown in Figure 3B,C: at the concentration of 5 mM, the complete separation of the analytes was accomplished. Under the same conditions (5 mM concentration), βCD showed a similar separation ability as well as the same migration pattern, with the exception of the D,L-Pro, which migrated as a peak pair between c3D/c3L and c4D/c4L enantiomer couples (Figure 4).

Interestingly, using both DM-βCD and βCD as the chiral selector, all the *cis* isomers migrated faster than *trans* derivatives; additionally, the D-enantiomers exhibited a faster apparent mobility than their corresponding L-enantiomers.

The migration order was the same using the two different CDs as chiral selectors, except for D-Pro, whose migration anticipated that of c4D in the system containing DM-βCD. The developed system showed an excellent chemo- and stereoselectivity with both βCD and DM-βCD. However, the latter provided a generally better resolution and a shorter analysis time and was selected for further investigations, method validations, and applications to real samples. The effect of the BGE concentration at a pH of 3.5 was evaluated in the range of 400–600 mM in the presence of DM-βCD at a 5 mM concentration. The increased BGE concentration significantly increased analysis times, while the separation was maintained under all the tested conditions. To limit the influence of the peak tailing of the reagent excess, while maintaining a short analysis time and better peak shape, the optimal conditions were considered as follows: the BGE constituted of a 500 mM sodium acetate at a pH of 3.5, supplemented with 5 mM DM-βCD (Figure 3C).

### 2.2. The Optimization of the Derivatization Conditions

Collagen hydrolysate contains the complete set of the proteinogenic amino acids, which, reacting with (R)-NCS, can interfere with the determination of 3- and 4-hydroxyprolines and D- and L-Pro. Since the target analytes are all secondary amino acids (also called imino acids) while the other amino acids released by the protein hydrolysis possess a primary amino group, it was possible to exploit their different reactivity by developing a two-step derivatization method. In the first step, the combined reagent ortho-phthalaldheyde/2-mercaptoethanol (OPA/ME) was used as it specifically reacts with primary amino functions to produce isoindole derivatives, while leaving the secondary amino acids unreacted. These could be derivatized in the second derivatization step, using the reagent (R)-NCS, to produce the corresponding thiourea derivatives detectable by LEDIF with an excitation wavelength at 480 nm, a condition under which the isoindole derivatives obtained in the first derivatization step do not interfere.

**Figure 5:**
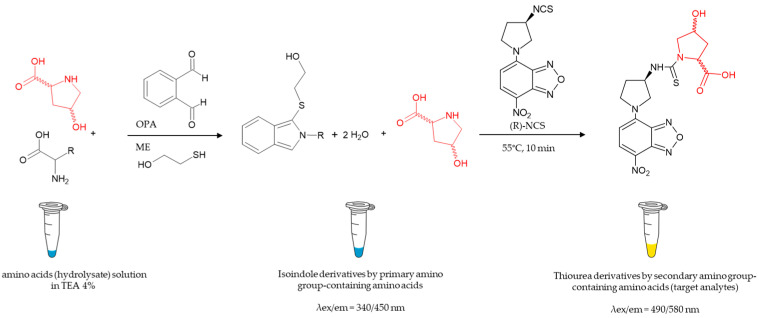
The scheme of the two-step derivatization and proposed structures [19,21] of the obtained derivatives. Abbreviations and symbols: OPA: ortho-phthalaldehyde, ME: 2-mercaptoethanol; and TEA: triethylamine. A generic 4-hydroxyproline molecule is depicted in red.

A scheme of the derivatization workflow, including the proposed structure of the obtained derivatives, is shown in Figure 5. The OPA/ME molar ratio has an important effect on the reaction course [21,22,23]; thus, the first step of the derivatization was optimized by testing different OPA/ME molar ratios (1/10, 1/5 and 1/2.5), while keeping the OPA/amino acids ratio constant at the value of six. A standard mixture of all the proteinogenic amino acids was prepared at a total concentration of about 10 mM to mimic the conditions of a protein hydrolysate, containing the secondary amino acids (the target analytes) each in the concentration range of 20–80 µM. The reaction was carried out in an alkaline medium to have the amino functions of the analytes as free bases, and it showed to be complete in a very short time (5 min) at room temperature when using an OPA/ME molar ratio of 1:2.5. The second derivatization step targeted the five enantiomeric couples, which, due to their secondary amino group, did not react with OPA/ME. While it has been reported that a [(R)-NCS/amino acids] molar ratio of 5 is adequate for the quantitative response of the analytes in standard solutions [19], in the present study the maximization of the response was achieved with a molar ratio [(R)-NCS/amino acids] ≥ 15. The observed behavior can be attributed to the inhibitory effect from the excess OPA/ME remaining from the first derivatization step. The reaction was found to be complete under mild conditions (55 °C for 10 min).

The LEDIF responses of the derivatives were comparable (*p* < 0.05, Table 1) whether proteinogenic primary amino acids were present or absent in the standard mixture. In addition, no interference by the latter was observed, demonstrating that under the described conditions the quantitation of the target analytes can be selectively and accurately carried out in real samples of collagen hydrolysates. The derivatization reaction was stopped by acidifying the mixture with acetic acid in a water/acetonitrile (ACN) solution to avoid the precipitation of the excess reagent. The use of acetic acid at high concentrations (e.g., 1 M in water/ACN, 50/50, *v*/*v*), as suggested for HPLC applications [19], produced broadened peaks. It was observed that reducing the acetic acid concentration improved the peak shape, likely due to a stacking phenomenon that was beneficial to minimize the peak tailing caused by the (R)-NCS excess reagent. The best conditions were achieved using 0.05 M of acetic acid in water/ACN (50/50, *v*/*v*), which allowed us to maintain a clear sample solution and to carry out the injection into the CE apparatus by the hydrodynamic mode at 50 mbar × 10 s, avoiding a current drop.

### 2.3. Method Validation

Linearity and sensitivity were evaluated in standard solutions, and the data obtained are shown in Table 2.

The sensitivity was about one order of magnitude lower than that obtained by GC-MS [11], a method that however did not resolve t4L from c4D. In our previous HPLC-MS study using extracted ion chromatograms, the analysis of a hydrolyzed chicken type II collagen sample revealed the presence of t4L, and, at lesser extents, t3L and c4D [9]. The observed levels were in the same order as those determined in the present study by the CE approach applied to similar chicken collagen samples. No other minor hydroxyproline enantiomers were detected using both the HPLC-MS and CE method, suggesting that the sensitivity of the latter, even if lower (less than one order), is adequate for the intended purpose. Compared to the chiral method for the separation of the stereoisomers of 4-hydroxyproline derivatized using the FMOC-Cl and UV detection [18], the present method showed a higher sensitivity, even though the last step of the procedure involves a 100-fold dilution of the initial collagen hydrolysate sample volume.

Recovery was assessed by spiking a representative sample of chicken type III collagen subjected to gas-phase hydrolysis with standard solutions of D-Pro, c4D, L-Pro, t4L, and t3L in the concentration ranges reported in Table 3. The other hydroxyproline isomers were not detected at the sensitivity limits of the applied methods and were therefore not considered in the recovery assessment. The recoveries ranged from 92.1 to 112.0%, in line with the values reported in previous studies based on the acidic hydrolysis of the collagen samples [8,9,11]. Based on three replicate analyses of the spiked sample, the RSD% values for the recovery determination were in the range of 4.2–7.6%. The good precision of the migration times (RSD% ≤ 2.5%, n = 6) allowed for the unambiguous identification of the analytes by comparing the migration time with those of standard compounds. The whole method precision was assessed by the determination of L-, D-Pro, and hydroxyproline enantiomers (c4D, t4L, and t3L) in six independent real samples (chicken type III collagen); the RSD% was lower than 7% [24]. In the time span of six hours, six replicated injections of the derivatized sample were performed; the RSD% values of the peak area for D-Pro, c4D, L-Pro, t4L, and t3L were 2.8%, 2.3%, 1.7%, 2.1%, and 2.8%, respectively. These results suggested that the sample solutions were stable for at least six hours at room temperature in the dark. The robustness of the method was assessed by quantifying the stereoisomers in the sample prepared for the recovery and precision evaluation method. The separation conditions applied in the robustness study were deliberately changed with respect to the optimum of some relevant parameters (acetate buffer 500 ± 10 mM, DM-βCD 5 ± 1 mM, cartridge temperature 25 ± 1 °C). The analyte concentration was within the confidence interval (α = 0.05; n = 3), demonstrating the robustness of the method.

### 2.4. Application to Chicken Collagen Hydrolysates

Several studies were conducted in the early 1980s to ascertain the implications of the onset of dystrophic conditions on chicken skeletal muscles’ ultrastructure and functionality [25,26]. In an intriguing study performed by DeMichele et al. it was shown that the proline hydroxylation in collagen extracted from the *gastrocnemius* muscle of embryonic chickens was lower in cases of muscular dystrophies. It was hypothesized that the consequent decrease in the inter- and intramolecular cross-linking would lead to a reduced stability of the collagen fibrils [27]. These conditions, that frequently occur in fast-growing birds, lead to remarkable modifications of the muscular architecture involving both the fibers (which exhibit a rounded cross-section and internal nuclei, along with the occurrence of necrotic processes up to the lysis and infiltrations of inflammatory cells) and the connective tissue composing the perimysial and endomysial septa [28].

**Figure 6:**
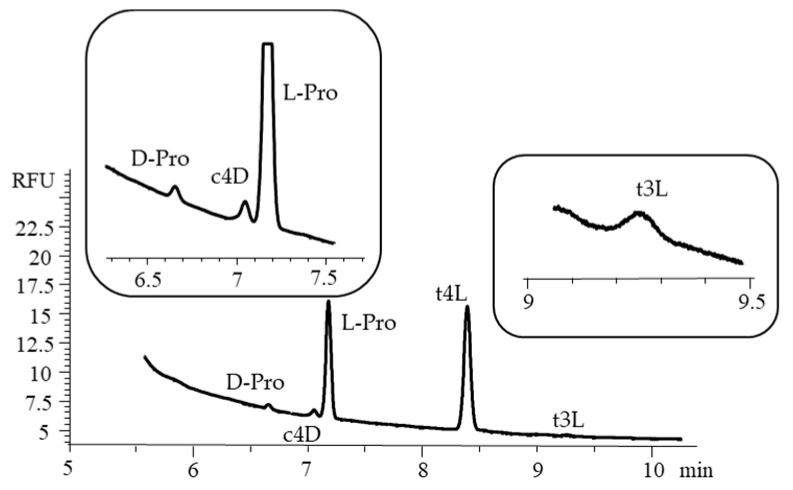
The electropherogram of a collagen type III hydrolysate extracted from chicken *Pectoralis major* muscle affected by a Wooden Breast abnormality; the insets provide magnified details of the electropherogram, highlighting the areas corresponding to the target analytes. Conditions and symbols as in Figure 3C.

Among the growth-related abnormalities observed in fast-growing chickens, the Wooden Breast (WB) and Spaghetti Meat (SM) conditions are those leading to the most dramatic changes at both the endo- and perimysial level. Histological and immunohistochemical observations performed on WB and SM muscles revealed that the first is characterized by an increased deposition of collagen (and connective tissue components), whereas the second manifests with its progressive rarefaction [29]. Among the different collagen types which could be found in skeletal muscle, collagen type III plays a relevant role in its embryonic development as well as during muscle regeneration [30]. In addition, differences in its amount and distribution were previously associated with the development of myopathic/dystrophic muscles and fibrotic processes as well [27]. Within this context, although several investigations have been carried out in the past two decades concerning the microscopic features of normal, WB, and SM muscles, information about the post-translational modifications occurring in collagen type III are still unknown. In the present application, collagen type III was isolated from the *Pectoralis major* muscle of fast-growing chickens considering macroscopically normal muscles as well as severe WB or SM cases. Collagen samples were subjected to gas-phase hydrolysis, and the proposed chiral CD-CZE method was applied to assess the distribution pattern and the quantitation of proline and hydroxyproline stereoisomers. The electropherogram related to the analysis of a collagen type III hydrolysate extracted from chicken *Pectoralis major* muscles affected by WB myopathy is shown in Figure 6.

The most represented compounds are L-Pro and t4L. The presence of a small amount of c4D, yielded by the partial isomerization of t4L during the gas-phase acid hydrolysis of the collagen type III extract, was observed [7,8,9]. The isomer t3L was also found at a very low amount, according to previous studies [9,31,32,33]. Interestingly, a small amount of D-Pro was detected, likely due to the racemization of L-Pro. This evidence is in agreement with the report by Opekar et al., who estimated the content of the proline distomer of 1.6–2.3% with respect to L-Pro, depending on the conditions of the acid hydrolysis of the collagen [11]. Even though only trace amounts of the distomers were produced through isomerization, they can significantly interfere with the accurate quantitation of the native eutomers if the chiral separation is not applied, highlighting the importance of the stereoselective determination. In Table 4 the distribution of the target compounds is reported, expressed as a percentage amount quantified using the specific calibration curves for each isomer in collagen type III hydrolysates obtained from the *Pectoralis major* muscles of broiler chickens exhibiting a macroscopically normal phenotype (N) as well as from those affected by SM and WB myopathies. Three samples were considered for each of the groups (i.e., N, WB, and SM) and were obtained by pooling nine independent samples per group, grouped three by three, thus resulting in composite samples (total of twenty-seven chickens).

While D-Pro and c4D could not be considered as useful biomarkers for the collagen characterization because of their artifactual origin, the distribution of the other target analytes was found to corroborate the previous report [27]. The number of samples analyzed is too limited to draw conclusions and for the in-depth analysis of post-translational modifications in type III collagen from chickens affected by myopathies. However, this study provides preliminary results showing that the sample preparation/derivatization and CD-CZE quantitation can be reliably applied for further investigations. Indeed, the level of t4L, the most represented hydroxylated isomer of L-proline, was shown to be higher, even if not significantly (*p* < 0.1), in collagen type III of N samples with respect to the ones extracted from the WB- and SM-affected muscles. These findings seem to corroborate the evidence of a previous study performed by Mazzoni et al. [28], in which the distributions of procollagen and collagen type III were evaluated in chicken *Pectoralis major* muscles—both unaffected cases and muscles showing evident signs ascribable to the WB and SM abnormality—through immunohistochemistry. These outcomes, together with the evidence of the present investigation, seem to suggest the presence of a more immature collagen type III in WB and SM muscles in comparison with the one found in N. This hypothesis may be further corroborated by considering that the development of hypoxic conditions, and hypoxia-mediated pathways, is associated with the onset and progression of the growth-related abnormalities in chickens. In fact, since proline hydroxylation is known to be an oxygen-dependent process, oxygen deprivation at the muscular level in fast-growing chickens may play a pivotal role in the post-translational modifications stabilizing collagen type III.

## 3. Materials and Methods

### 3.1. Materials

(2S,4R)-4-hydroxypyrrolidine-2-carboxylic acid (*trans*-4-hydroxy-L-proline, t4L), was from Merck KGaA (Darmstadt, Germany). (2R,3S)-3-hydroxypyrrolidine-2-carboxylic acid hydrochloride (*cis*-3-hydroxy-D-proline, c3D) and (2S,3S)-3-hydroxypyrrolidine-2-carboxylic acid (*trans*-3-hydroxy-L-proline, t3L) were provided by Ambeed (Arlington Hts, IL, USA). (2R,3R)-3-hydroxypyrrolidine-2-carboxylic acid (*trans*-3-hydroxy-D-proline, t3D) and (2S,3R)-3-hydroxypyrrolidine-2-carboxylic acid (*cis*-3-Hydroxy-L-proline, c3L) were from Biosynth (Staad, Switzerland). (2R,4S)-4-hydroxypyrrolidine-2-carboxylic acid (*trans*-4-hydroxy-D-proline, t4D) and (2R,4R)-4-hydroxypyrrolidine-2-carboxylic acid (*cis*-4-hydroxy-D-proline, c4D) were from ThermoFisher Scientific (Waltham, MA, USA), while (2S,4S)-4-hydroxypyrrolidine-2-carboxylic acid (*cis*-4-hydroxy-L-proline, c4L) was from TCI Europe (Zwijndrecht, Belgium). The standard amino acids were the following glycine (Gly), glutamine (Gln), arginine (Arg), serine (Ser), glutamic acid (Glu), aspartic acid (Asp), threonine (Thr), alanine (Ala), L-proline (L-Pro), D-proline (D-Pro), methionine (Met), valine (Val), phenylalanine (Phe), isoleucine (Ileu), leucine (Leu), histidine (His), lysine (Lys), hydroxylysine (HLys), and cysteine (Cys), and all of them were purchased from Sigma-Aldrich (Milan, Italy).

The derivatization addressed to the light-emitting diode-induced fluorescence (LEDIF) detection was carried out by using (R)-(-)-4-(3-isothiocyanatopyrrolidin-yl)-7-nitro-2,1,3-benzoxadiazole ((R)-NCS), by TCI Europe (Zwijndrecht, Belgium). The derivatization of the primary amino acids was carried out by the reagents o-phthalaldehyde (OPA) and 2-mercaptoethanol (ME) purchased by Sigma-Aldrich (Milan, Italy). The following cyclodextrins were used as additives to the background electrolyte (BGE): β-cyclodextrin (βCD), γ-cyclodextrin (γ-CD), (2-hydroxypropyl)-β-cyclodextrin (HP-βCD), heptakis(2,6-di-O-methyl)-β-cyclodextrin (DM-βCD), and heptakis(2,3,6-tri-O-methyl)-β-cyclodextrin (TM-βCD)—all purchased by Sigma-Aldrich (Milan, Italy).

The chemicals used to perform the gas-phase hydrolysis of the collagen samples and for the preparation of BGE buffers, e.g., hydrochloric acid, sodium hydroxide, dimethyl sulfoxide, acetic acid, sodium tetraborate, triethylamine (TEA), methanol, and acetonitrile (ACN), were from Sigma-Aldrich (Milan, Italy).

Collagen type III samples were obtained according to the procedure described by DeMichele et al. [27], which is based on limited pepsin extraction followed by collection of the digested collagen type III through selective salt precipitation. Then, collagen type III was purified by means of ion-exchange chromatography on DEAE-cellulose column.

Ultrapure water was used for the preparation of running buffers, samples, and standard solutions and was purified by Elix Systems (Millipore, Billerica, MA, USA).

### 3.2. Solutions

Standard stock solutions of amino acids (about 1 mg/mL) were prepared in water and stored at 2–8 °C for one week. Working standard solutions were prepared by appropriate dilution with a mixture of water/ACN (50/50, *v*/*v*) containing 4% (*v*/*v*) TEA.

The derivatization reagent mixture OPA/ME was prepared at the concentration of 28/70 mM; in detail, an aliquot of 250 μL of a methanol solution of OPA (15 mg/mL) was mixed with 200 μL tetraborate buffer (30 mM) and 5 μL of ME. The volume was completed to 1 mL with methanol. The reagent (R)-NCS was prepared at the concentration of 10 mM in ACN. All the solutions were stored at 2–8 °C for one week.

### 3.3. Gas-Phase Hydrolysis of Collagen Samples

Gas-phase hydrolysis of the collagen samples was carried out as described previously [8,9] with some modifications: collagen samples were dissolved in water with 0.1% acetic acid at a concentration of 1 mg/mL. Next, 100 μL aliquots of the resulting solution were placed into small glass inserts and dried under a nitrogen stream. Subsequently, each insert was placed into a glass tube containing 300 μL of 6 M HCl and hermetically sealed; the tubes were then incubated in an oven at 110 °C for 24 h. Any remaining HCl in the glass insert was removed under nitrogen, and the dried hydrolysate was reconstituted with 250 μL of a mixture water/ACN (5/0/50, *v*/*v*) containing TEA at 4%.

### 3.4. Derivatization Reactions

The derivatization procedure of the standard mixture or the real samples from gas-phase hydrolysis was undertaken in two steps: (i) Aliquots of 10 μL of the sample (or standard) solution were mixed with 20 μL of OPA/ME reagent solution; the mixture was vortexed and kept at room temperature for 5 min. (ii) Next, 60 μL of the reagent (R)-NCS solution was added and vortexed, then it was warmed at 55 °C for 10 min. The reaction was stopped by adding an acetic acid solution of 0.05 M in water/ACN (50/50, *v*/*v*) to the final volume of 1.0 mL.

### 3.5. CE Instrumentation and Analysis

The instrument for CE analysis was a G1600 HP3DCE from Agilent Technologies (Waldbronn, Germany) using the software Rev. A. 09. 01. Agilent Chemstation. Fused-silica capillaries (CM Scientific, Dublin, Ireland) were 55.5 cm total length and 34 cm to the detector, with the inner diameter (i.d.) of 50 μm (outer diameter 363 μm). New capillaries were conditioned by flushing in the following order: sodium hydroxide 1M, sodium hydroxide 0.1M, and water (10 min each). The analysis under optimized conditions was performed by using a BGE composed of acetate buffer of 500 mM (pH 3.5), supplemented with 5 mM of DM-βCD. Between the injections, the washing/conditioning cycle of the capillary was as follows: 2 min sodium hydroxide 0.1 M, 1 min dimethyl sulfoxide/water (50/50, *v*/*v*), 4 min water, and 3 min BGE. The injection of the sample was performed by hydrodynamic mode at 50 mbar for 10 s. The applied voltage was constant at 30 kV, and the cartridge temperature was set at 25 °C. Detection was performed using the ZetaLIF LED detector (LED-Induced Fluorescence Detector, LEDIF) by Adelis SAS (Labège, France), with 480 nm excitation wavelength and collecting the fluorescence emission in the range of 515–760 nm.

### 3.6. CE Method Validation

Linearity was assessed for all the hydroxyprolines isomers and D-Pro in the range of 1–100 μM (in the presence of a constant amount of 80 μM of L-Pro, to mimic the real sample conditions) and 20–200 μM, for L-Pro and t4L. Sensitivity was estimated by progressive dilution of standard solutions of the analytes; the limit of detection (LOD) value was determined as the concentration providing a signal-to-noise ratio (S/N) = 3 and the limit of quantitation (LOQ) at S/N = 10. The noise was assumed as the distribution of the response at zero analyte concentration. The instrumental precision was assessed as degree of repeatability of migration time and peak area (RFU, relative fluorescence response); the method precision was estimated by the quantitation of the analytes in the same spiked samples of the recovery study as the RSD% (n = 6) [24].

Recovery tests were performed by quantifying D-Pro, c4D, L-Pro, t4L, and t3L in three independent samples (n = 3) spiked into chicken type III collagen hydrolysates. From the same samples, the recovery repeatability was also calculated. The robustness study was carried out by quantifying the found analytes in a representative sample (chicken type III collagen) using CE conditions involving parameters which were separately changed over an established range around their optimized values. In detail, the concentration of the BGE constituted of acetate buffer was varied within 500 ± 10 mM; the concentration of DM-βCD was varied within 5 ± 1 mM; and the temperature of the cartridge was varied within 25 ± 1 °C.

## 4. Conclusions

The proposed cyclodextrin-modified capillary electrophoresis method enabled the fast separation of all the four enantiomeric pairs of hydroxylated proline as well as that of D,L-proline, useful biomarkers in the compositional analysis of collagen. The method exhibits an exceptional stereoselectivity achieved through the synergistic combination of the target analytes’ derivatization with a chiral reagent and the incorporation of cyclodextrins as chiral selectors within the electrophoretic buffer. The critical role of cyclodextrins in attaining the complete resolution of the analytes was demonstrated, underscoring the tunability of the separation by the proper selection of the cyclodextrin type and concentration. The optimized and validated system provides an orthogonal analytical tool to chromatographic methods and has demonstrated its utility in the quantification of proline and hydroxyproline isomers in collagen hydrolysates, serving as potential biomarkers for myopathies in chickens.

## Figures and Tables

**Table 1:** LEDIF responses ^(1)^ of the secondary amino acid derivatives in the standard solution (response 1) and in the presence of the complete set of the proteinogenic amino acids (response 2).

Amino Acid ^(2)^	Response 1 ± sd ^(3)^	Response 2 ^(4)^ ± sd ^(3)^
c3D	0.509 ± 0.02	0.548 ± 0.02
c3L	1.00 ± 0.04	0.937 ± 0.02
D-Pro	0.221 ± 0.01	0.194 ± 0.01
c4D	0.639 ± 0.03	0.678 ± 0.02
L-Pro	0.232 ± 0.01	0.208 ± 0.01
c4L	0.956 ± 0.03	0.940 ± 0.03
t4D	1.02 ± 0.04	0.928 ± 0.04
t4L	1.05 ± 0.03	1.12 ± 0.03
t3D	0.658 ± 0.03	0.738 ± 0.04
t3L	0.995 ± 0.04	1.08 ± 0.04

^(1)^ RFU, relative fluorescence signal using LEDIF detector at the excitation wavelength of 480 nm; ^(2)^ concentration ranging within 20–80 μM; abbreviations as in Figure 1; ^(3)^ n = 3. ^(4)^ The total concentration of the mixture of the primary amino acids is about 10 mM.

**Table 2:** Linearity and sensitivity data (in µM).

Amino Acid	Range, μM	Equation ^(1)^	r^2^	LOD	LOQ ^(2)^
c3D	1–100	y = 0.3240x − 0.0147	0.999	0.08	0.20
c3L	1–100	y = 0.2051x − 0.0361	0.998	0.08	0.20
D-Pro	1–100	y = 0.0643x + 0.0168	0.998	0.20	0.50
c4D	1−100	y = 0.2264x + 0.0513	0.999	0.08	0.20
L-Pro	20–200	y = 0.0416x − 0.0506	0.999	0.20	0.50
c4L	1–100	y = 0.1727x − 0.0949	0.998	0.10	0.30
t4D	1–100	y = 0.1900x − 0.0316	0.999	0.10	0.30
t4L	20–200	y = 0.2033x − 0.0251	0.999	0.10	0.30
t3D	1–100	y = 0.1938x + 0.0185	0.998	0.15	0.40
t3L	1−100	y = 0.3119x + 0.0327	0.999	0.15	0.40

^(1)^ x concentration in µM; ^(2)^ RSD% ≤ 6; n = 3.

**Table 3:** Recovery data.

Amino Acid	Content ^(1)^ ± sd	Spike ^(1)^	Recovery% (RSD%)
D-Pro	2.51 ± 0.12	3.0	105.4 (5.5)
c4D	2.56 ± 0.17	3.0	92.1 (6.7)
L-Pro	66.6 ± 2.1	80.0	94.6 (5.6)
t4L	89.2 ± 2.8	80.0	93.5 (4.2)
t3L	0.80 ± 0.07	1.0	112.0 (7.6)

^(1)^ Concentration in µM.

**Table 4:** The distribution as the percentage of the target analytes in collagen type III extracted from Normal (N), Spaghetti Meat (SM), and Wooden Breast (WB) chicken Pectoralis major muscles (n = 3/group, results are expressed as mean ± sd).

Amino Acid	SM	WB	N
D-Pro	1.21 ± 0.23	0.98 ± 0.37	0.68 ± 0.35
c4D	1.66 ± 0.22	1.25 ± 0.26	1.08 ± 0.19
L-Pro	39.6 ± 2.2	40.5 ± 2.54	38.7 ± 2.6
t4L	55.8 ± 2.2	56.6 ± 2.3	59.3 ± 1.9
t3L	0.95 ± 0.11	0.59 ± 0.19	0.65 ± 0.14

## Data Availability

All the data can be found in the main body of the article.

## Abbreviations

The following abbreviations are used in this manuscript:

CE	Capillary Electrophoresis
DM-βCD	Heptakis(2,6-di-O-methyl)-β-cyclodextrin
L-Pro	L-Proline
D-Pro	D-Proline
t4L	*trans*-4-hydroxy-L-proline
t3L	*trans*-3-hydroxy-L-proline
t4D	*trans*-4-hydroxy-D-proline
t3D	*trans*-3-hydroxy-D-proline
c4L	*cis*-4-hydroxy-L-proline
c3L	*cis*-3-hydroxy-L-proline
c4D	*cis*-4-hydroxy-D-proline
c3D	*cis*-3-hydroxy-D-proline
HPLC	High-Performance Liquid Chromatography
MS	Mass Spectrometry
GC-MS	Gas Chromatography Mass Spectrometry
BGE	Background Electrolyte
CD	Cyclodextrin
MEKC	Micellar Electrokinetic Chromatography
CZE	Capillary Zone Electrophoresis
CD-CZE	Cyclodextrin-modified Capillary Zone Electrophoresis
FMOC-Cl	9-fluorenylmethylchloroformate
(R)-NCS	(R)-(-)-4-(3-Isothiocyanatopyrrolidin-yl)-7-nitro-2,1,3-benzoxadiazole
LEDIF	light-emitting diode-induced fluorescence
β-CD	β-cyclodextrin
EOF	Electroosmotic flow
γCD	γ-cyclodextrin
HP-βCD	(2-hydroxypropyl)-β-cyclodextrin
TM-βCD	heptakis(2,3,6-tri-O-methyl)-β-cyclodextrin
OPA	ortho-phthalaldheyde
ME	2-mercaptoethanol
TEA	Triethylamine
ACN	Acetonitrile
RFU	Relative Fluorescence Unit
LOD	Limit of Detection
LOQ	Limit of Quantitation
SM	Spaghetti Meat myopathy
WB	Wooden Breast myopathy
